# Cholesterol-Lowering Effects of Asperidine B, a Pyrrolidine Derivative from the Soil-Derived Fungus *Aspergillus sclerotiorum* PSU-RSPG178: A Potential Cholesterol Absorption Inhibitor

**DOI:** 10.3390/ph15080955

**Published:** 2022-07-31

**Authors:** Atcharaporn Ontawong, Acharaporn Duangjai, Yaowapa Sukpondma, Kwanruthai Tadpetch, Chatchai Muanprasat, Vatcharin Rukachaisirikul, Jakkapong Inchai, Chutima S. Vaddhanaphuti

**Affiliations:** 1 Division of Physiology, School of Medical Sciences, University of Phayao, Phayao 56000, Thailand; atcharaporn.on@up.ac.th (A.O.); achara.phso@gmail.com (A.D.); 2 Division of Physical Science and Center of Excellence for Innovation in Chemistry, Faculty of Science, Prince of Songkla University, Songkhla 90112, Thailand; yaowapa.suk@psu.ac.th (Y.S.); kwanruthai.t@psu.ac.th (K.T.); vatcharin.r@psu.ac.th (V.R.); 3 Chakri Naruebodindra Medical Institute, Faculty of Medicine Ramathibodi Hospital, Mahidol University, Bangkok 10540, Thailand; chatchai.mua@mahidol.ac.th; 4 Department of Physiology, Faculty of Medicine, Chiang Mai University, Chiang Mai 50200, Thailand; jakkapong.inc@gmail.com

**Keywords:** cholesterol absorption, LXRα, NPC1L1, pyrrolidine derivative, asperidine B (preussin)

## Abstract

Isolated secondary metabolites asperidine B (preussin) and asperidine C, produced by the soil-derived fungus *Aspergillus sclerotiorum* PSU-RSPG178, were found to exhibit inhibitory effects against 3-hydroxy-3-methyl-glutaryl-coenzyme A reductase and oxidative stress in an in vitro assay. Whether or not the known pyrrolidine asperidine B and the recently isolated piperidine asperidine C have lipid-lowering effects remains unknown. Thus, this study aimed to investigate the hypocholesterolemic effects of asperidines B and C and identify the mechanisms involved in using in vitro, ex vivo, and in vivo models. The results show that both compounds interfered with cholesterol micelle formation by increasing bile acid binding capacity, similar to the action of the bile acid sequestrant drug cholestyramine. However, only asperidine B, but not asperidine C, was found to inhibit cholesterol uptake in Caco-2 cells by up-regulating LXRα without changing cholesterol transporter NPC1L1 protein expression. Likewise, reduced cholesterol absorption via asperidine-B-mediated activation of LXRα was also observed in isolated rat jejunal loops. Asperidine B consistently decreases plasma cholesterol absorption, similar to the effect of ezetimibe in rats. Therefore, asperidine B, the pyrrolidine derivative, has therapeutic potential to be developed into a type of cholesterol absorption inhibitor for the treatment of hypercholesterolemia.

## 1. Introduction

An imbalance of lipid levels, including hypercholesterolemia- and hypertriglyceridemia-induced obesity, has been known to become a major risk factor for several diseases, including type 2 diabetes mellitus, cardiovascular disease, hypertension, cancer, and non-alcoholic fatty liver disease [1,2,3]. Therefore, the control of cholesterol homeostasis in the body is thought to be an important factor in preventing obesity-related diseases. Cholesterol in the body comes from two major sources: 30% of total cholesterol is from dietary intake while 70% of cholesterol is synthesized in the body, particularly in the liver [4]. It has been revealed that dietary lipids in the form of cholesterol micelles are absorbed into enterocytes by the lipid uptake transporter Niemann-Pick C1-Like 1 (NPC1L1). This transporter plays a critical role in cholesterol absorption mediated by a clathrin-coated endocytosis mechanism on the apical membrane of the proximal jejunum and canalicular membrane of hepatocytes in humans and rodents [5,6].

Studies have previously reported that a lack of intestinal NPC1L1 resulted in a reduction in plasma cholesterol and low-density lipoprotein cholesterol (LDL-C) and improved fatty liver in high-cholesterol-diet-fed mice, while intestinal NPC1L1 expression increased in cholesterol-depleted-diet-fed porcine [7,8]. Furthermore, activation of liver X receptor alpha (LXRα) by LXR agonists T0901317 and GW3965 down-regulated NPC1L1, reducing cholesterol absorption in Caco-2 cells and a mouse model [9,10]. A clinical study also reported that single polymorphisms of the NPC1L1 gene have been associated with future cardiovascular events in coronary disease patients [11]. Prescription of ezetimibe, an NPC1L1 inhibitor, for a period of 3 months, reduced plasma LDL-C by 18.58% in hypercholesterolemic patients [11]. In addition, ezetimibe combined with simvastatin decreased the rate of cardiovascular events in patients with acute myocardial infarction [12]. Thus, intestinal NPC1L1 has a significant impact on the progression of hyperlipidemia and related diseases. Although statins are recommended as a first-line drug against hyperlipidemia and obesity-associated diseases, non-statins including ezetimibe, proprotein convertase subtilisin/Kexin type 9 inhibitor, and bempedoic acid have been considered for nonresponsive individuals [13].

**Figure 1:**
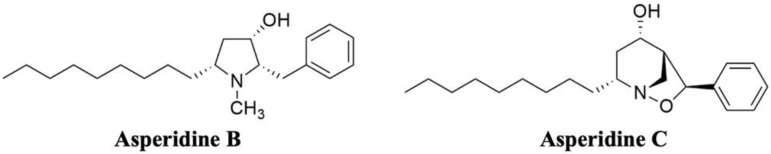
Structures of asperidine B (preussin) and asperidine C isolated from the soil-derived fungus *Aspergillus sclerotiorum* PSU-RSPG178.

In recent years, soil-derived fungi have been gaining increasing attention due to their production of various bioactive secondary metabolites [14]. Among these, crude extracts of the soil-derived fungus *A. sclerotiorum* PSU-RSPG178 have shown antimalarial and anti-mycobacterial activity [15], while purified lovastatin and its analogue, α,β-dehydromonocolin S, exhibited a lipid-lowering effect against 3-hydroxy-3-methylglutaryl-coenzyme A reductase activity and hepatic steatosis in high-fat-diet-induced obese rats [15,16]. Moreover, our study also found antioxidant activity of the secondary metabolite asperidine B (preussin) and asperidine C, produced by the same fungus, *A. sclerotiorum* PSU-RSPG178, isolated from a soil sample collected from the Plant Genetic Conservation Project under the Royal Initiation of Her Royal Highness Princess Maha Chakri Sirindhorn at Ratchaprapa Dam in Suratthani Province, Thailand [15,17]. Asperidine B has recently become known as an alkaloid (+)-preussin [17], whereas asperidine C is a recently isolated piperidine derivative (Figure 1). A recent study reviewed how a piperidine moiety exerts several pharmacological activities including anticancer, antiviral, antimalarial, antifungal, antihypertension, anti-Alzheimer’s, and anti-inflammatory activities [18]. A previous study reported that preussin has antitumor activity by inducing apoptosis in human cancer cell lines [19]. In addition, pyrrolidine dithiocarbamate, a potent antioxidant and inhibitor for nuclear factor-kappa B activation, shows hepatoprotective effects against liver injury induced by lipopolysaccharide and ischemia/reperfusion in rodents [20,21,22]. This compound also alleviates multiple organ failures induced by zymosan [23]. Moreover, pyrrolidine dithiocarbamate exhibits antidiabetic effects in both obese and lean Zucker rats [24]. Similarly, pyrrolidine analogues display potent antidiabetic and lipid-lowering activity by acting as peroxisome proliferator-activated receptor (PPAR) α/γ agonists in both in vitro and diabetic db/db mice [25]. Thus, besides their antioxidant activity, it is unknown whether asperidine B (preussin) and asperidine C exhibit lipid-lowering activity. Therefore, this study aimed to investigate whether asperidine B (preussin) and asperidine C have lipid-lowering effects using in vitro, ex vivo, and in vivo models. The cellular and molecular mechanisms of these compounds in reducing lipid levels were also determined.

## 2. Results

### 2.1. Asperidines B and C Interfere with the Physicochemical Properties of Cholesterol Micelles by Binding with Secondary Bile Acid

**Figure 2:**
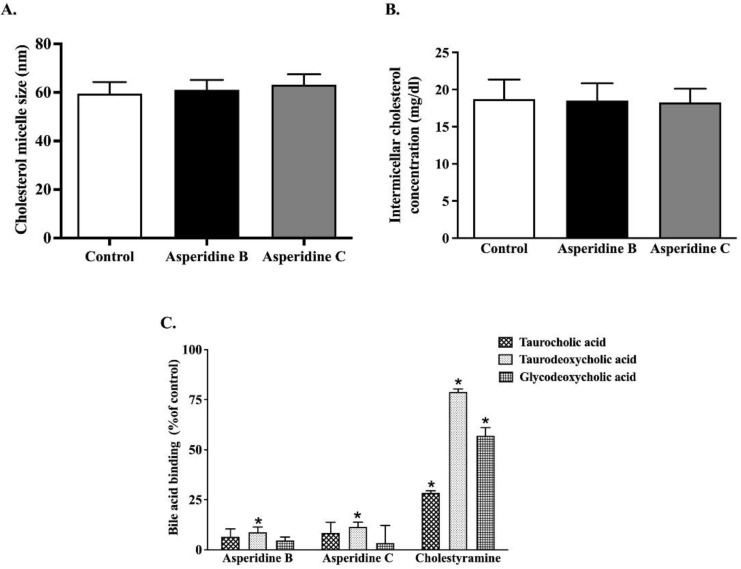
Effects of asperidine B (preussin) and asperidine C on (**A**) micellar cholesterol particle size, (**B**) intermicellar cholesterol levels, and (**C**) bile acid binding capacity. Values are mean ± SEM (*n* = 3), * *p* < 0.05 vs. control.

Since the formation of cholesterol micelles is a critical step for intestinal cholesterol absorption, particularly that which is mediated by NPC1L1 [5], we determined the following physicochemical properties of cholesterol micelles exposed to both asperidines B and C: cholesterol micelle particle size, cholesterol solubility, and bile acid binding capacity. As shown in Figure 2A,B, neither compound influenced micelle particle size or cholesterol solubility. On the other hand, their affinity for binding with a secondary bile acid, taurodeoxycholic acid, was statistically significant when compared with the control. Nonetheless, they had no capacity to bind with the primary bile acid, taurocholic acid, and secondary bile acid, glycodeoxycholic acid. This suggests that both asperidines B and C have potential for inhibiting intestinal cholesterol absorption, partly by interfering with bile acid binding capacity, thereby leading to reduced cholesterol micelle formation and absorption.

### 2.2. Asperidine B, but Not Asperidine C, Decreases Cholesterol Absorption in Intestinal Caco-2 Cells

**Figure 3:**
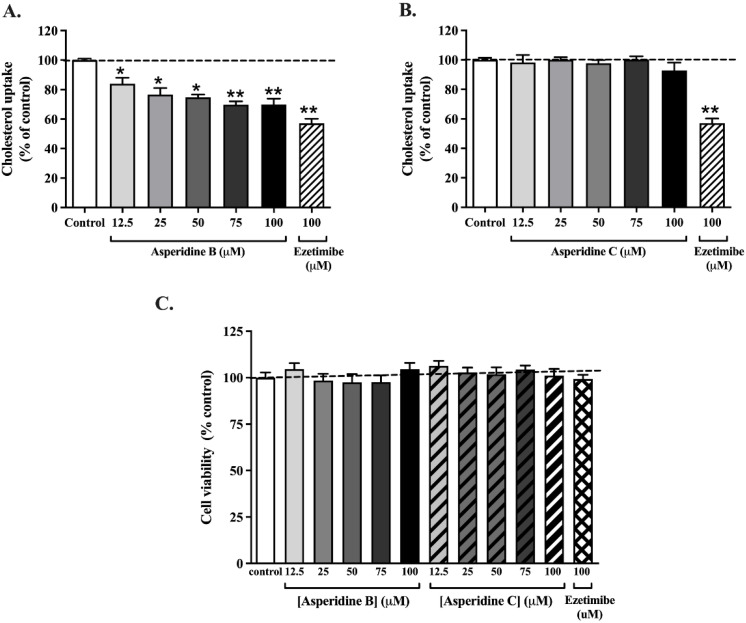
Effect of asperidine B (preussin) and asperidine C on [^3^H]-cholesterol mixed micelle transport in intestinal Caco-2 cells. (**A**) Asperidine B and (**B**) asperidine C at 12.5–100 µM or 100 µM ezetimibe were co-incubated with 1 μCi/mL [^3^H]-cholesterol mixed micelles for 30 min at 37 °C (*n* = 5). (**C**) Viability of CacO-2 cells after exposure to asperidine B, asperidine C, or ezetimibe at the same concentrations as shown in (**A**,**B**). Caco-2 cells were pre-incubated with tested compounds for 3 h and incubated with an MTT reagent. Absorbance was measured at a wavelength of 570 nm and percentage cell viability was compared with control cells. Data are represented as mean ± SEM (*n* = 5); * *p* < 0.05, ** *p* < 0.01 vs. control.

As shown in Figure 3A, asperidine B at 12.5–100 µM significantly reduced [^3^H]-cholesterol mixed micelle transport in a concentration-dependent manner when compared with the control. Notably, at higher concentrations of asperidine B (75 and 100 μM), there was a similar degree of inhibition to that elicited by 100 μM ezetimibe, an NPC1L1 inhibitor. On the other hand, asperidine C had no effect on [^3^H]-cholesterol mixed micelle transport (Figure 3B). To confirm if the reduction in cholesterol transport was due to cytotoxicity, cell viability under exposure to both compounds was also examined. As shown in Figure 3C, asperidines B and C at 12.5–100 µM, as well as 100 µM ezetimibe, had no effect on the viability of intestinal Caco-2 cells. This indicates that asperidine B efficiently inhibits cholesterol absorption in human intestinal Caco-2 cells similar to ezetimibe, a cholesterol absorption inhibitor.

### 2.3. Asperidine B Up-Regulates LXRα Expression without Alteration of NPC1L1 Membrane Protein Expression in Intestinal Caco-2 Cells

**Figure 4:**
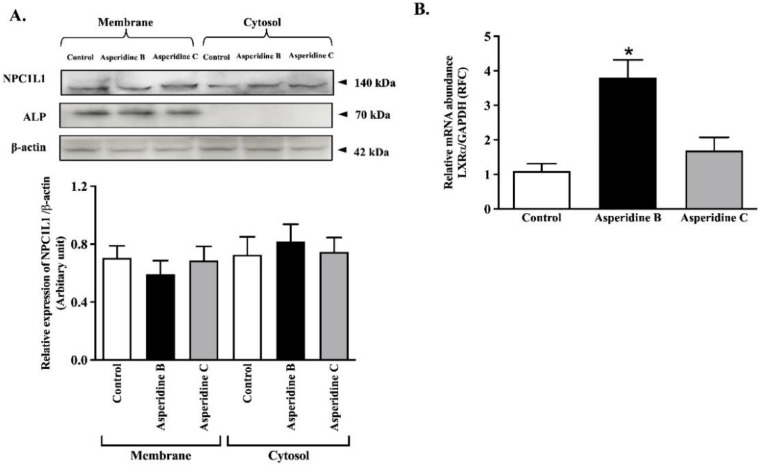
Expression of NPC1L1 and LXRα in Caco-2 cells. Cells were incubated with 100 µM asperidines B and C for 3 h. (**A**) Membrane and cytosolic fractions expressing NPC1L1 were detected by electrophoresis and a Western blot analysis. Anti-alkaline phosphatase (ALP) and anti-β-actin antibodies were used as a membrane marker and loading control, respectively. A representative blot of NPC1L1 protein expression is shown in the top panel and quantification of relative NPC1L1/β-actin protein expression in each fraction is presented at the bottom. (**B**) Total RNA was extracted from Caco-2 cells and LXRα mRNA levels were determined using qPCR. Data are expressed as mean ± SEM from five separate experiments. Data are represented as mean ± SEM (*n* = 3); * *p* < 0.05 vs. control.

To further investigate the mechanism by which asperidine B reduced [^3^H]-cholesterol mixed micelle transport by down-regulating the NPC1L1 membrane transport protein, fractions of membrane and cytosolic proteins were extracted from Caco-2 cells and the NPC1L1 protein was subsequently analyzed using Western blotting. Although the enrichment of membrane and loading control proteins was indicated by the expression of ALP and β-actin, neither asperidines B nor C altered NPC1L1 expression in the membrane and cytosolic compartments (Figure 4A). LXRα is known to be a negative regulator of NPC1L1 by inducing internalization of NPC1L1 [9]. As expected, asperidine B up-regulated LXRα mRNA expression while asperidine C had no effect (Figure 4B). This implies that asperidine B partly activates the LXRα signaling pathway and might interfere with NPC1L1 function, leading to reduced cholesterol absorption in intestinal Caco-2 cells.

### 2.4. Asperidine B Strongly Inhibits Cholesterol Transport by Activation of LXRα in Rat Jejunal Loops

**Figure 5:**
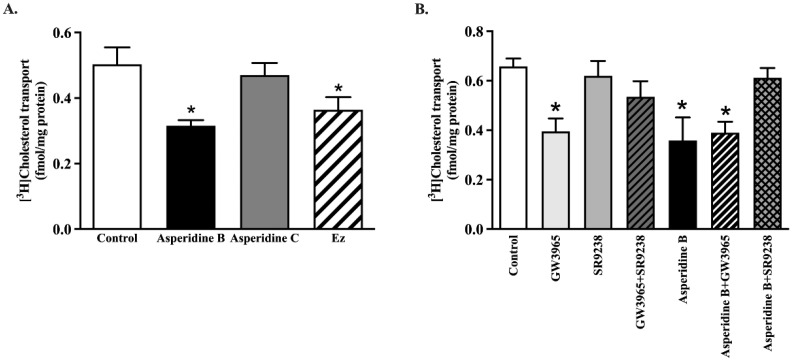
Effect of asperidines B and C, ezetimibe, LXRα activator (GW3965), and LXRα inhibitor (SR9238) on [^3^H]-cholesterol transport using ex vivo rat jejunal loops: (**A**) 100 µM asperidine B, asperidine C, or ezetimibe was co-injected in rat jejunal loops and incubated for 30 min; (**B**) 100 µM asperidine B, 1 µM LXRα activator (GW3965), 10 µM LXRα inhibitor (SR9238), a combination of GW3965 with either SR9238 or asperidine B, and a combination of SR9238 with asperidine B were incubated in a buffer containing [^3^H]-cholesterol mixed micelle for 30 min. The radioactivity accumulated in intestinal epithelium was then measured and expressed as fmol/mg protein. Values are mean ± SEM (*n* = 6), * *p* < 0.05 vs. control.

To confirm whether asperidines B or C inhibited cholesterol uptake in intact tissues, we measured [^3^H]-cholesterol micelle uptake in rat jejunal loops. As seen in Caco-2 cells, transport of [^3^H]-cholesterol mixed micelles decreased by approximately 37% on treatment with 100 µM asperidine B when compared with the control, while asperidine C had no effect (Figure 5A). When adding 100 µM ezetimibe, cholesterol mixed micelle transport was affected to the same extent as by asperidine B. In addition, activation of LXRα by 1 µM GW3965, an LXRα agonist, reduced cholesterol absorption in jejunal epithelial cells compared with the control, while 10 µM SR9238, an LXRα antagonist, reversed cholesterol uptake to a normal level (Figure 5B). The effect of the LXRα agonist also counteracted its antagonist in the GW3965 + SR9238 group. Consistently, cholesterol transport was significantly decreased by asperidine B, whereas GW3965 restored asperidine B’s effect, leading to restoration of cholesterol transport back to a normal level (Figure 5B). The data strongly confirm that asperidine B (preussin) inhibits intestinal cholesterol absorption mediated by LXRα activation.

### 2.5. Asperidine B Acts as a Cholesterol Absorption Inhibitor by Reducing Plasma Cholesterol Levels in Rats

**Figure 6:**
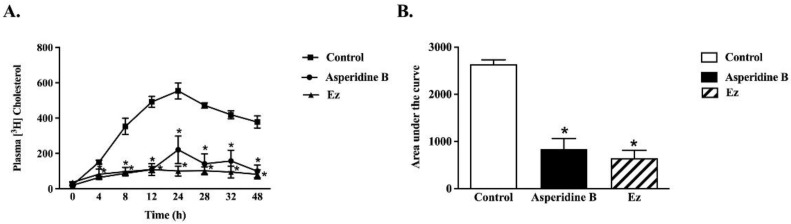
Effect of asperidine B or ezetimibe on plasma [^3^H]-cholesterol in rats. (**A**) The plasma [^3^H] cholesterol level was measured at 0, 4, 8, 12, 24, 32, and 48 h after oral administration with [^3^H]cholesterol (10 µCi/mL), with or without 31.75 µg/kg BW asperidine B or 10 mg/kg BW ezetimibe. (**B**) The total area under the curve from (**A**) was analyzed. Values are mean ± SEM (*n* = 4–6), * *p* < 0.05 vs. control.

To thoroughly confirm the inhibitory effect of asperidine B on intestinal cholesterol absorption, the plasma [^3^H]-cholesterol level was quantified. As shown in Figure 6A, administration of asperidine B at 31.75 µg/kg body weight (BW), which is approximately equal to 100 µM used in the ex vivo model, markedly reduced plasma [^3^H]-cholesterol levels throughout the 48 h of the experiment when compared with the control rats. This finding was similar to the treatment of 10 mg/kg BW of ezetimibe. The calculated area under the curve also consistently showed that asperidine B significantly reduced the plasma [^3^H]-cholesterol level to the same extent as ezetimibe (Figure 6B), strongly suggesting that asperidine B inhibits intestinal cholesterol absorption.

## 3. Discussion

This study is the first to demonstrate the promising effects of a pyrrolidine derivative, asperidine B (preussin), but not a recently isolated piperidine derivative, asperidine C, in lowering cholesterol by inhibiting cholesterol absorption in in vitro, ex vivo, and in vivo models. This pyrrolidine interference with bile acid binding capacity results in reduced cholesterol micellar formation and absorption. Asperidine B also exhibits a high potency, similar to that of the prescribed drug, ezetimibe, mediated by LXRα activation, which could subsequently interfere with NPC1L1 function. Since cholesterol homeostasis depends upon two major sources—receipt from the diet and de novo cholesterol synthesis—the former is approximately proportional to intestinal cholesterol absorption by contributing roughly 50% of total plasma cholesterol [26]. Several clinical studies suggest that a high plasma cholesterol level is a major risk factor for atherosclerosis, cardiovascular diseases, and a risk of death [27,28]. Hence, intestinal cholesterol absorption is a primary and critical step for intervention targeting.

At present, ezetimibe is known to be the first and only prescribed lipid-lowering drug for the inhibition of dietary cholesterol absorption, which is crucial for statin-intolerant patients [29]. It has been shown that ezetimibe monotherapy decreases total cholesterol, LDL-C, apolipoprotein B, and non-high density lipoprotein cholesterol levels in hyperlipidemia patients [30]. More recently, the benefit of combining ezetimibe with statins has been extensively studied. It has been shown that acute coronary syndrome (ACS) patients, with a baseline LDL-C exceeding 131 mg/dL who receive ezetimibe added to pitavastatin, have better clinical outcomes when compared with ACS patients receiving monotherapy by statin [31]. In addition, ezetimibe and simvastatin reduce cardiovascular events after ACS in both non-diabetic and diabetic patients [32]. In contrast, coadministration of ezetimibe and another lipid-lowering drug, such as cholestyramine, might cause drug interaction [30]. Thus, any compounds acting similar to the mechanism of ezetimibe’s action could be of benefit and warrant increased attention in drug discovery and development.

Although neither asperidines B nor C altered cholesterol micelle size and solubility, both compounds have primarily shown their ability to bind to taurodeoxycholic acid, the secondary bile acid in this study. Therefore, this inaccessible bile acid could influence cholesterol micelle formation and absorption. As such, the balance of bile acids and sterol compositions are critical factors for intestinal lipid absorption, reducing bile acid flow and the size of the bile acid pool through this enterohepatic circulation, simultaneous with the new synthesis of hepatic cholesterol to bile acids, which, in turn, reduces the whole body’s cholesterol level [33]. Likewise, oat bran with native β-glucan induces bile acid and cholesterol excretion in the gut, together with the possibility of entrapping bile acids and interfering with micellar structures, resulting in reduced cholesterol absorption in patients with well-functioning ileostomies [34]. Our previous study suggested that coffee pulp extract (CPE) not only increases bile acid binding capacity similar to cholestyramine, but also interferes with the physicochemical characteristics of micellar cholesterol, including increased cholesterol solubility and cholesterol micelle particle size, leading to lower cholesterol levels in both Caco-2 cells and hypercholesterolemic rats [35]. An increase in the size of micellar cholesterol by epigallocatechin gallate and *Azadirachta indica* leaf extract is also reflected in cholesterol absorption [36,37].

Inhibition of intestinal cholesterol absorption in Caco-2 cells and jejunal epithelium by asperidine B, but not asperidine C, was strongly demonstrated in this study. There are currently several possible mechanisms to lower cholesterol absorption by either reproposing drugs or by natural products, which have been extensively studied. Down-regulation of NPC1L1 has apparently been shown to be a dominant factor for reducing intestinal cholesterol absorption. For example, lycopene has been shown to activate LXRα, resulting in decreased NPC1L1 expression and function in Caco-2 cells [38]. LXR activation by its agonist, T0901317, also down-regulates NPC1L1 mRNA expression, resulting in reduced cholesterol absorption in both mice and a human enterocyte cell line [9]. The evidence also shows that the LXRα agonist GW3965 inhibits the development of atherosclerosis in both *Ldlr^−/−^* and *Apoe^−/−^* mice [39], indicating that the LXRα activator has an atheroprotective effect. As with ezetimibe, directly binding with NPC1L1 remains a key mechanism for reducing cellular cholesterol uptake into the intestinal epithelium [40]. Orlistat, a known lipase inhibitor, is suggested to be a non-competitive inhibitor for NPC1L1 by decreasing cholesterol uptake by 30% [41]. In this study, even though asperidine B (preussin) did not change the membrane expression of NPC1L1, it has been clearly proven to exhibit 30–37% inhibition of cholesterol absorption mediated by LXRα activation in an in vitro model, Caco-2 cells, and rat jejunal loops, and to have a higher efficacy, as seen in vivo (68% inhibition). Nonetheless, a limitation of this study is the lack of clarity as to whether NPC1L1 is a direct target for asperidine B. In fact, kinetic parameters of ezetimibe, represented by a reduced maximum transport rate (Vmax) without alteration of the affinity for binding with NPC1L1 (Km; the concentration of substrate that is transported at half the Vmax), could refer to either changes in NPC1L1 membrane expression or its translocation in Caco-2 cells [42,43]. Since asperidine B (preussin) inhibits cholesterol absorption at less than 50%, the apparent kinetic parameters of asperidine B mediated by NPC1L1 could hardly be demonstrated in this study. Our previous study also clearly suggested that reduced cholesterol absorption by CPE was revealed by down-regulation of NPC1L1 membrane protein expression, while NPC1L1 internalization was suggested by kinetic parameters: in other words, a reduced Vmax without changing the Km [35]. Therefore, the promising action of asperidine B could be due to activation of LXRα, which may interfere with NPC1L1 function and, in turn, lower cholesterol levels. Although the overall cholesterol-lowering activities of CPE in our previous study [35] and asperidine B (preussin) in this study were similar to those of ezetimibe, there are stark differences in the molecular mechanisms between these two compounds. CPE could non-competitively bind to NPC1L1 and decrease its expression on the membrane, together with changing the physicochemical properties of micelle cholesterol, resulting in reduced cholesterol absorption [35]. On the other hand, asperidine B differentially activates LXRα and may subsequently interfere with NPC1L1 function while simultaneously binding to bile acid. It can be expected that either binding, interfering with NPC1L1 function, or changing the physicochemical properties of micelle cholesterol, regardless of any compound, would result in a similar outcome of effective reduction in plasma cholesterol levels. As previously mentioned, oxybenzyl pyrrolidine acid acts as a PPARα/γ agonist in both binding and functional assays as well as exerting a potent anti-triglyceridemic effect in diabetic db/db mice [25]. Likewise, asperidine B contains a pyrrolidine core similar to that of oxybenzyl pyrrolidine acid. Thus, this structural moiety might contribute to the cholesterol-lowering activity mediated by activating PPARs. PPARα activated by fenofibrate has consistently been revealed to be another negative regulator of NPC1L1 [44] similar to LXRα, while the agonist of PPARδ, GW1516, is associated with reduced NPC1L1 mRNA expression [45]. Taken together, this notion leads us to further identify molecular targets in mechanisms of the cholesterol-lowering effects of asperidine B (preussin). In addition, advanced identification strategies, such as chemical proteomic approaches, together with mass spectrometry and bioinformatic technologies, have been highlighted and could be a future challenge; therefore, it is important to have an insightful understanding of the mechanisms of intestinal cholesterol absorption by asperidine B (preussin).

## 4. Materials and Methods

### 4.1. Chemicals

Asperidines B and C (>98% purity by NMR analysis) used in this study were isolated from the soil-derived fungus *Aspergillus sclerotiorum* PSU-RSPG178 as previously described [17]. Dulbecco’s modified Eagle’s medium (DMEM/F12), unlabeled cholesterol, phosphatidylcholine, sodium taurocholate, and CelLytic MT mammalian tissue lysis/extraction reagents were obtained from Sigma Aldrich (St. Louis, MO, USA). Fetal bovine serum (FBS) was purchased from Gibco (Carlsbad, CA, USA). Polyclonal rabbit anti-NPC1L1 and monoclonal mouse alkaline phosphatase (ALP) antibodies were purchased from Novus Biologicals (Littleton, CO, USA). Monoclonal mouse anti-β actin was purchased from Abcam (Cambridge, MA, USA). [^3^H]-cholesterol, specific activity 49 Ci/mmol, was obtained from Perkin Elmer (Waltham, MA, USA). A complete protease inhibitor cocktail was purchased from Merck (Darmstadt, Germany). All other chemicals with high purity were obtained from commercial sources.

### 4.2. Determination of Physicochemical Properties of Micellar Complex

As previously described [35], the effects of asperidines B and C on micelle particle size, micelle solubility, and bile acid binding capacity have been determined. Micelles were prepared by mixing 3 μM unlabeled cholesterol, 6 mM sodium taurocholate, and 0.15 mM phosphatidylcholine and evaporated by N_2_ gas. The sample was reconstituted by sonication in phosphate buffered saline (PBS) and passed through 0.22 µm syringe filters. Asperidine B or C at 100 μM was then added into the mixed micelle solution and incubated for 3 h at 37 °C. The size of the micellar complex was subsequently measured using a particle size analyzer (Malvern Instruments Ltd., Malvern, UK). Similarly, to determine the micellar cholesterol, 10 mM unlabeled cholesterol, 1 mM sodium taurocholate, and 0.6 mM phosphatidylcholine were mixed in PBS. The mixed micelle solution was incubated in the presence or absence of 100 μM asperidines B or C for 3 h at 37 °C. The micelles were filtered through a 0.22 µm membrane to separate precipitated micelles from intermicellar complex. The filtrated micellar cholesterol was measured using a commercial colorimetric cholesterol assay (Biotechnical Co., Ltd., Bangkok, Thailand). Furthermore, we investigated the interference of bile acid binding capacity by asperidines B or C and compared it with that of cholestyramine, a bile acid sequestrant drug. Briefly, asperidines B or C at 100 μM was incubated with either 2 mM taurocholic acid, glycodeoxycholic acid, or taurodeoxycholic acid at pH 7.0, 37 °C for 2 h. The bile acid mixture was centrifuged at 10,000 rpm for 10 min, and the supernatant was filtered through a 0.22 µm membrane filter to separate bound and free bile acids. The filtrated free bile acid was mixed with a reaction mixture containing 0.133 M tris buffer at pH 9.5, 1 M hydrazine hydrate, 7.7 mM nicotinamide adenine dinucleotide (NAD), and 1 unitmL/3α-hydroxysteroid dehydrogenase. The reaction mixture was further incubated at 37 °C for 2 h. The rate of thio-NADH formation was measured at a wavelength of 340 nm and calculated as a percentage of the control.

### 4.3. Preparation of Cholesterol Mixed Micelles

A cholesterol mixed micelle solution was prepared for the cholesterol transport study by mixing 1 µCi/mL [^3^H]-cholesterol, 2 µM unlabeled cholesterol, 0.1 mM phosphatidylcholine, and 4 mM sodium taurocholate. The mixture solution was evaporated with N_2_ gas and reconstituted by sonicating in serum-free DMEM/F12 for 1 h. The cholesterol mixed micelle solution was subsequently filtered through a 0.22 µm syringe filter prior to use in the cholesterol transport study.

For the in vivo cholesterol absorption experiment, the micelle solution containing 10 µCi/mL [1α,2α(n)-^3^H]-cholesterol, 1 µM unlabeled cholesterol, 50 µM phosphatidyl-choline, and 2 mM sodium taurocholate was mixed and evaporated with N_2_ gas and stored at −20 °C until use. The cholesterol micelles were freshly prepared by dissolving the lipid film in PBS and sonicating for 1 h. The cholesterol micelle solution was subsequently filtered through a 0.22 µm syringe filter and maintained at 37 °C before use.

### 4.4. Cell Culture

The human colorectal adenocarcinoma (Caco2) cell line was purchased from the American Type Culture Collection (Manassas, VA, USA). Cells in the 2nd–22nd passage were grown in DMEM/F12 containing 20%/FBS and 1% penicillin–streptomycin in a humidified atmosphere containing 5% CO_2_. Caco-2 cells were seeded in 24- and 96-well plates at a density of 5.0 × 10^4^ cells/well and were cultured at 37 °C for 18–21 days to become a differentiated Caco-2 monolayer. The medium was replaced every 3 days during culturing.

### 4.5. Cholesterol Transport in Human Intestinal Caco-2 cells

Differentiated Caco-2 cells were washed three times with PBS (pH 7.4) and incubated with 1 μCi/mL [^3^H]-cholesterol mixed micelles in the presence or absence of 12.5–100 µM asperidines B and C for 3 h. The medium was removed, and the cells were washed three times with ice-cold PBS. The cells were subsequently dissolved in 1 N NaOH and neutralized by 1 N HCl. The radioactivity of cholesterol transport was measured using liquid scintillation counters (Perkin Elmer, MA, USA). The intracellular [^3^H]-cholesterol micelle radioactivity was calculated as fmol/mg protein/min.

### 4.6. Determination of Cell Viability

The viability of Caco-2 cells after exposure to tested compounds was determined using the 3-(4,5-dimethylthiazol-2-yl)-2,5-diphenyltetrazolium bromide (MTT) assay. Caco-2 cells were seeded at a density of 5.0 × 10^4^ cells/well in 96-well plates and cultured for 18–21 days at 37 °C. On the day of the experiment, the culture medium with or without asperidines B or C was added and incubated for 3 h at 37 °C. After exposure, the cells were washed with PBS, the culture medium containing MTT reagent was added, and the resulting mixture was incubated for the next 4 h at 37 °C. At the end of the experiment, the MTT solution was aspirated, and the cells were washed once with ice-cold PBS. DMSO was added to each well and incubated for another 30 min at 37 °C. The absorbance of dissolved formazan was measured at a wavelength of 570 nm using a Synergy^TM^ HT microplate reader (Agilent Biotek, Santa Clara, CA, USA). The absorbance of the sample detected at a wavelength of 680 nm was also used as a reference.

### 4.7. Quantitative Real-Time PCR Analysis

Total RNA was extracted and purified from Caco-2 cells using a TRIzol reagent (Thermo Fisher Scientific, MA, USA) according to the manufacturer’s instructions. First-strand cDNA was obtained using an iScript cDNA synthesis kit (Bio-Rad, Hercules, CA, USA) and qPCR was performed using an SYBR real-time PCR master mix (Bioline, London, UK) on an ABI 7500 Fast system (Life Technologies, NY, USA). Forward and reverse primers were purchased from Macrogen (Seoul, Korea) and used at a final concentration of 0.4 μM. The specific primer set for human LXRα and glyceraldehyde 3-phosphate dehydrogenase (GAPDH) is as follows: hLXRα (forward primer: 5′-AAGCCCTGCATGCCTACGT-3′, reverse primer: 5′-TGCAGACG CAGTGCAAACA-3′) and human GAPDH (forward primer: 5′-AGCCTTCTCCATGGTGGTGAAGAC-3′, reverse primer: 5′-CGGAGTCAACGGATTTGGTCG-3′). Gene expression was normalized to GAPDH mRNA levels and reported as relative fold changes (RFC). QPCR amplification was performed in duplicate for each cDNA.

### 4.8. Subcellular Fractionation and Western Blot Analysis

To measure the membrane protein expression of cholesterol transporter NPC1L1, subcellular fractions were prepared using differential centrifugation. Caco-2 cells were lysed using a lysis buffer containing a 1% complete protease inhibitor mixture according to the manufacturer’s protocol. Briefly, samples were disrupted by a homogenizer and centrifuged at 5000× *g* for 10 min at 4 °C. The supernatant was designated as a whole cell lysate. Half of the supernatant was re-centrifuged at 100,000× *g* for 2 h. The supernatant fraction from this step was designated as the cytosolic fraction, while the pellet was re-suspended with the same buffer and used as the membrane fraction. The protein concentration in each sample was also determined using the Bradford assay (Bio-Rad, CA, USA), and samples were stored at −80 °C prior to use. For the Western blot analysis, samples were resolved in 4× Laemmli solution, electrophoresed on 10% SDS-PAGE, and transferred onto polyvinylidene difluoride (PVDF) membranes (GE Healthcare, Chicago, IL, USA). Non-specific binding was eliminated by blocking with 5% *w*/*v* non-fat dried milk in 0.05% Tween-20 in a tris-buffered saline (TBS-T) for 1 h and incubated overnight with either polyclonal anti-rabbit NPC1L1, monoclonal anti-mouse ALP, or anti-mouse β-actin antibodies. The PVDF membrane was washed with TBS-T buffer and incubated with horseradish-peroxidase-conjugated ImmunoPure secondary goat, anti-rabbit, or anti-mouse IgG (Merck, Darmstadt, Germany) for 1 h. Proteins were detected using the SuperSignal West Pico Chemiluminescent Substrate (GE Healthcare, WI, USA) and quantitatively analyzed using the ImageJ program from the Research Services Branch (RSB) of the National Institute of Mental Health (NIMH; Bethesda, MD, USA).

### 4.9. Ex vivo and In Vivo Cholesterol Transport Measurements

Male Wistar rats weighing 300–350 g were obtained from Nomura Siam International (Bangkok, Thailand). The animal facilities and protocols were approved by the Laboratory Animal Care and Use Committee at the Faculty of Medicine, Chiang Mai University, Chiang Mai, Thailand (protocol no: 17/2564 and 03/2565). All the rats were housed in a room maintained at 25 ± 1 °C on a 12:12 h dark–light cycle and acclimated for 1 week with free access to chow and water. To address the effect of asperidines B and C on cholesterol transport using isolated jejunal loops as previously described [35], small intestinal tracts were removed, cleaned, and flushed with PBS. The jejunum was ligated to a length of 1.5 cm and intraluminally injected with PBS containing 1 µCimL/ [^3^H]-cholesterol mixed micelles in the presence or absence of asperidine B, asperidine C, or ezetimibe. The jejunal loop was then incubated in PBS buffer for 30 min at room temperature. The PBS buffer was also collected after 10, 20, and 30 min to ensure leakage of the [^3^H]-cholesterol throughout incubation. To identify mechanisms involved with the NPC1L1 function, LXRα activator (GW3965) 1 µM or LXRα inhibitor (SR9238) 10 µM were replaced either on their own or combined with a tested compound. At the end of the experiment, the jejunal loop was cut horizontally and the epithelial tissue was removed. The jejunal epithelium was weighed, dissolved in 1 N NaOH, and neutralized by using 1 N HCl. The intracellular [^3^H]-cholesterol radioactivity was measured using liquid scintillation counters (Perkin Elmer, Waltham, MA, USA), and calculated as fmol/mg protein/min.

To further determine actual cholesterol absorption in an in vivo model, the animals fasted for 12–16 h. The blood [^3^H]-cholesterol level was measured at 0 min as a baseline. Subsequently, 10 µCi/mL [^3^H]-cholesterol micelles containing either the tested compound or ezetimibe were orally administered, and the blood samples were collected at 4, 8, 12, 24, 28, 32, and 48 h from the tail vein. Plasma radioactivity was analyzed by liquid scintillation spectroscopy (Perkin Elmer, MA, USA).

### 4.10. Statistical Analysis

Data were expressed as mean ± SEM. Statistical differences were assessed using one-way ANOVA followed by Tukey’s post hoc test. Statistical analyses were conducted using the statistical package for IBM SPSS Statistical Software version 23 (IBM Co., Armonk, NY, USA). Differences were considered to be significant when *p* < 0.05.

## 5. Conclusions

This study demonstrates the promising and high-efficacy cholesterol-lowering action of a pyrrolidine derivative, asperidine B (preussin), by reducing intestinal cholesterol absorption, mediated by the activation of LXRα and interference with bile acid binding capacity. Therefore, asperidine B (preussin) containing a pyrrolidine core has great potential to be developed as a cholesterol absorption inhibitor for the treatment of hypercholesterolemia. The identification of molecular targets and understanding the mechanisms of action of asperidine B (preussin) warrants further investigation.

## Data Availability

Data is contained within the article.
